# A Global Analysis of the Relationship between Farmed Seaweed Production and Herbivorous Fish Catch

**DOI:** 10.1371/journal.pone.0148250

**Published:** 2016-02-19

**Authors:** E. James Hehre, Jessica J. Meeuwig

**Affiliations:** 1 Sea Around Us Project /Institute for oceans and Fisheries, University of British Columbia, Vancouver, BC, Canada; 2 School of Animal Biology and Centre for Marine Futures, University of Western Australia, Crawley, WA, Australia; Aristotle University of Thessaloniki, GREECE

## Abstract

Globally, farmed seaweed production is expanding rapidly in shallow marine habitats. While seaweed farming provides vital income to millions of artisanal farmers, it can negatively impact shallow coral reef and seagrass habitats. However, seaweed farming may also potentially provide food subsidies for herbivorous reef fish such as the Siganidae, a valuable target family, resulting in increased catch. Comparisons of reef fish landings across the central Philippines revealed that the catch of siganids was positively correlated to farmed seaweed production whilst negatively correlated to total reef fish catch over the same period of time. We tested the generality of this pattern by analysing seaweed production, siganid catch, and reef fish catch for six major seaweed-producing countries in the tropics. We hypothesized that increased seaweed production would correspond with increased catch of siganids but not other reef fish species. Analysis of the global data showed a positive correlation between farmed seaweeds and siganids in Southeast Asia (Indonesia, Malaysia, and the Philippines) but not Africa (Tanzania and Zanzibar), or the Western Pacific (Fiji). In Southeast Asia, siganid catch increased disproportionately faster with seaweed production than did reef fish catch. Low continuity, sporadic production and smaller volumes of seaweed farming may explain the differences.

## Introduction

The commercial cultivation of seaweeds occurs in approximately 35 countries around the world and provides a variety of products that, in 2011, produced 21 million tonnes with a total annual value of US$7.35 billion [1]. Of that total, food products contributed almost US$ 5 billion [1]. Seaweed cultivation continues to expand rapidly as demand for seaweed products such as carrageenan has outstripped supply from wild resources. Although seaweed farming occurs globally, the vast majority of seaweed farming (98.9%, 18.9 million tonnes) is concentrated in China (60%) and Southeast Asia, including Indonesia (21%), the Philippines (9%), and Malaysia (1%). Throughout Southeast Asia, small subsistence farms (<1 ha) predominate, and their proliferation is in large part governed by both accessibility to useable habitats and proximity to markets [2]. Rising demand for seaweed products and the need for impoverished communities to develop alternative livelihoods are driving seaweed farms to expand into new locations, including onto coral reefs [2,3].

The majority of the world’s coral reefs are found in developing countries with high rates of population growth [4]. Combined with social inequality, population growth has significantly increased pressures on tropical marine fisheries [2,5–8], which are an important source of revenue and protein for millions of people globally [5,9,10]. The ecological impacts of coastal population growth primarily derive from both the system loading effects of pollution and siltation and the extractive and degrading effects of resource overexploitation [11–14]. In many places, these impacts are exacerbated by the use of destructive practices like blast and cyanide fishing. Once damaged, the capacity of a reef to recover depends on several factors, including its fundamental starting condition and the degree to which the causes of reef decline have been removed. While a growing number of studies indicate that reef recovery is possible with effective implementation of coastal management and alternative livelihood programs [15–17], progress will be constrained unless human population growth rates slow and poverty is alleviated.

The benefits of seaweed farming are unclear despite the practice being advocated as a way to improve reef health through poverty alleviation and reduced fisheries exploitation [2]. Recent studies have shown that the introduction of seaweed farming does little to mitigate the effects of fisheries overexploitation, and that rather than replacing fishing, it is utilized as an additional source of income [18]. However, seaweed farms also tend to be located in easily accessible, shallow and sheltered habitats situated in close proximity to markets. As such, many of the areas in which farms are located have already been degraded by overfishing and habitat loss [19,20] and thus may not cause additional habitat degradation. However, the direct ecological impacts of seaweed farming are still debated as empirical studies have typically produced different and conflicting results. For instance, in Indonesia, Blankenhorn (2007) found that where seagrass was not cleared as part of farm establishment, seaweed farming itself had no negative effect on seagrass beds [21]. By contrast, Ekloff (2006) recommended that seaweed farming in shallow seagrass areas should be avoided and that damage to seagrass beds was mitigated only by the small scale of farms and the recovery periods dictated by generally low market prices [22].

Hehre and Meeuwig (2015) also showed that seaweed farming on degraded shallow coral reefs corresponded with lower species richness, abundance, and biomass of associated fish assemblages, despite initial speculation that farms may benefit fish assemblages by adding physical complexity and shelter, and a potential food source for herbivores [2,22–25]. These results are consistent with other studies that have shown both lower fish abundances and species richness in macroalgal-dominated versus coral-dominated habitats [26,27]. Furthermore it has been demonstrated experimentally that herbivorous fishes will avoid areas of high macroalgal biomass [28]. However, despite these findings, it is still possible seaweed farms increase rabbitfish productivity rather than standing biomass [29–31]. While the Underwater Visual Census methods used in Hehre & Meeuwig 2015 give us a measure of abundance, abundance may not necessarily reflect productivity [32,33]. Increases in siganid productivity relative to farming could be masked by the concentration of fishing effort within the farms.

Though they may provide benefits in terms of both recruitment and food, specific fisheries benefits derived from seaweed farms also remain unclear. Herbivorous fish such as siganids forage on a broad range of algae [34,35] [1,36–39]. Field studies have demonstrated that siganids play an important role as consumers of naturally-occurring macroalgae on coral reefs, and *Sargassum* in particular [36,40,41]. Additionally, siganid foraging on macroalgae has been blamed for wide-scale damage to seaweed crops throughout Southeast Asia [20].

Moreover, Hehre and Meeuwig (in review) found that although siganids feed on farmed seaweeds, the consumption of farmed seaweeds likely functions as a replacement for wild seaweeds rather than an actual subsidy to catches, given animals were smaller and less abundant within farmed areas than in areas not associated with farms. There is evidence that some species of siganids such as *Siganus canaliculatus*, *Siganus fuscescens* and *Siganus spinus* settle directly to macroalgal beds despite most siganids settling to coral-dominated habitats[26,34]. Many of the species that settle to macroalgal beds are also those that are frequently targeted by fishers, as is the case in the Philippines for *S*. *canaliculatus*. However, the potential of seaweed farms to enhance fisheries requires further investigation. In the context of declining fish returns and the potential for critical income for poor people, seaweed farms may be beneficial to reef fish by adding both structure to habitats homogenized by human presence and a potential food source to the environment. Additionally, in those areas already subjected to a high degree of disturbance, where the majority of substrate has already been negatively affected, the presence of any additional human disturbance may not be detectable. Indeed, it is possible that the addition of further human activities may in fact serve to benefit the underlying reef by reducing some of the most destructive activities like blast and cyanide fishing, and replacing them with less destructive ones. Limiting the structural degradation of reefs caused by destructive practices is particularly important in light of the link between coral reef decline and losses in fisheries productivity [42].

Here, we test the hypothesis that increased seaweed production is correlated with higher catches of siganids and whether siganid catches increase proportionately more quickly with seaweed production than associated reef fish catches. We use regional data from a major centre of seaweed farming in the Philippines on seaweed production and catches of the siganid, *S*. *canaliculatus*, to determine if a localised relationship exists and how this corresponds to reef fish catches more generally. We then collated global data on farmed seaweed and fisheries catches as reported to the FAO [1,2]. We focused on six tropical countries from three regions that both produce seaweeds and report siganid catches: Southeast Asia including Indonesia, Malaysia, and the Philippines; Africa including Tanzania, and Zanzibar; and the Western Pacific which was represented by Fiji. Combined, these regions account for 35% of global farmed seaweed production in 2011. We also extracted FAO catch data for non-herbivorous reef fish to determine whether siganid catches increased more quickly with increasing seaweed production, than general reef fish catches. These data allow us to understand how the large-scale implementation of seaweed farming throughout the tropics may influence catches of herbivores like siganids.

## Methods

### Local data

Bohol, a province located in the central Philippines, is a populous region with a substantial degree of poverty [2,3,18]. Residents of Bohol, particularly in the northern region of Danajon Bank, are highly dependent on both fishing and seaweed farming since few alternative income-generating opportunities exist [4,18,43–45]. Indeed, Danajon Bank is a major producer of farmed seaweed in the Philippines [2,5–8,45]. The majority of farming here is conducted on an artisanal scale: households are able to claim an area of up to 1 ha for farming on the reef which they are required to register with municipal agricultural officer [5,9,10,18]. Local subsistence fisheries are multispecies with a wide range of targets exploited for either direct consumption or sale, with siganids a key target for both purposes. Continued unsustainable levels of fishing effort combined with the use of illegal and destructive fishing methods, such as dynamite and cyanide, have lead to declining catches further compounding poverty (Green et al. 2000, 2004; Christie et al. 2006; Armada et al. 2009). Additionally the continued degradation of the reef structure from anthropogenic activities has lead Danajon bank to be classified as one of the most degraded reef systems in the world [11–14,46].

Seaweed production, and siganid and reef fish catch data for Bohol were extracted from the database of the Philippine Bureau of Agricultural Statistics (PBAS; [5,47]). The PBAS generates basic data on fisheries production and socioeconomic data related to agriculture and fisheries. Fisheries landings of reef fishes (in tonnes per year) were monitored for the years 2002–2012 as part of a government project designed to evaluate artisanal catch returns, and centered mainly on Danajon Bank [2,47].

### Regional data

**Fig 1:**
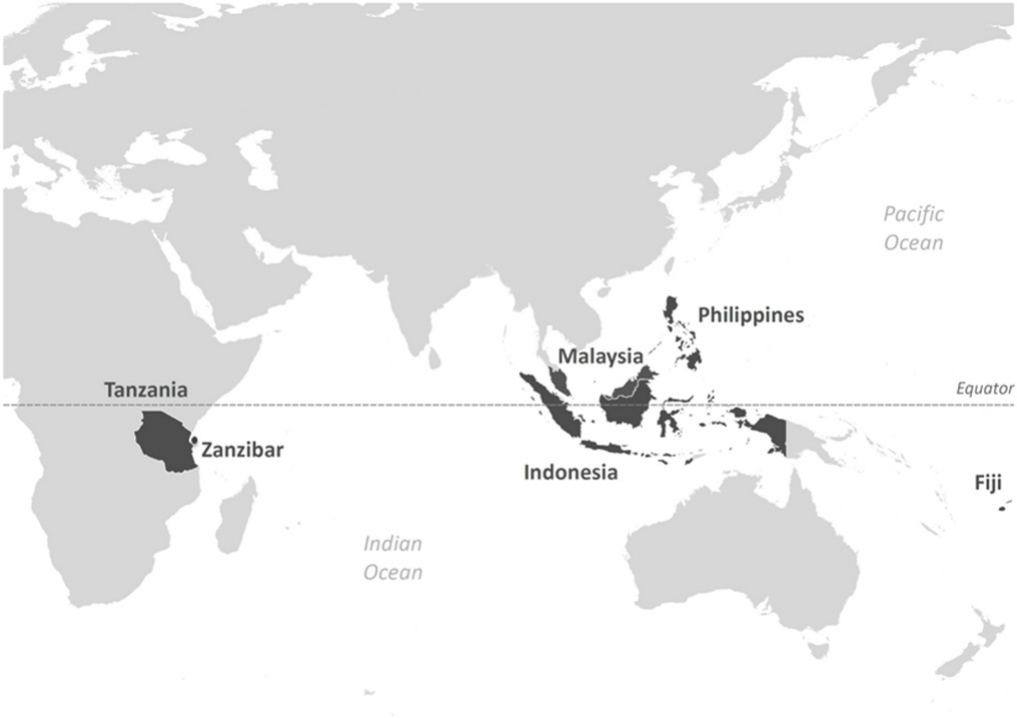
Countries from the three regions (Southeast Asia, Africa, and the Western Pacific) included in the global analysis of seaweed production.

Regional data on seaweed production and catches of herbivorous siganids and common reef fish species (in tonnes per year) have been compiled by the FAO since 1950 in varying levels of detail. We extracted these data for six countries in three regions: Southeast Asia (Indonesia, Malaysia, and the Philippines); Africa (Tanzania, and Zanzibar); and Western Pacific (Fiji) (Fig 1). A range of reef-associated taxa was selected (excluding siganids) as a control for trends in siganid catches, as effort data is unavailable. The inclusion of as many reef-associated species as possible was important in order to integrate the effects of changes in effort across a variety of reef fisheries independent of the gear used for extraction, which can vary within and among regions.

Combined, these countries account for 35% of the world’s seaweed production, and 58.4% when China is excluded. These countries were included in the analysis because concurrent records were available for seaweed production, siganid catches and reef fish catches for at least 15 years (FAO world fisheries and aquaculture 2012). Data were extracted from the Food and Agriculture Organisation of the United Nations (FAO) database using the Fishstat J software (http://www.fao.org/fishery/statistics/software/fishstatj/en). These statistics mainly represent commercial operations as artisanal, subsistence, and recreational fisheries are not typically reported [18,48,49]. As such production levels may underestimate total landings, depending on the scale of non-commercial activities.

Our analysis of commercially farmed seaweeds included all carrageenophytes, the marine plants commonly known as red seaweeds (Rhodophyceae). The carrageenan produced by these algae is a polysaccharide used as a hydrocolloid for the manufacture of many food, pharmaceutical and industrial products. Carrageenophytes comprise nearly 50% of global landings and receive the highest prices. The carrageenophytes in the FAO database are classified as “*Eucheuma* seaweeds nei” (where nei is “not elsewhere identified”) and “Spiny eucheuma” (interpreted as *Eucheuma spinosum*), *Gracilaria* red seaweeds (*Gracilaria* spp.), and elkhorn moss (*Kappaphycus alvarezii*).

Catch data were compiled for the siganids, a group of herbivores reported to FAO as “spinefeet”. Fisheries catches can increase simply due to increased effort through time independent of total abundance [19,20,50–52]. However, no effort data are available in the FAO database against which changes in catch could be controlled. As a surrogate control for effort, we also extracted the catch data for a range of reef fish species (excluding siganids) representing a total of 35 different families [S1 Table] to allow us to determine whether there was a disproportionate increase in siganid catches relative to catches of other reef fish, likely subject to similar levels of fishing effort and gears.

### Modelling

We used regression analysis to examine the relationship between siganid catches and seaweed production, and between siganid catches and reef fish catches. For each variable, we calculated the percentage of the maximum value (PMV) for each year as the fraction of the highest value observed over the time series. This was done for both fish catches and seaweed production in order to generate a general trend independent of volume [21,53]. Specifically, this then allows a comparison of a standardised change in siganid catch or reef fish catch as a function of a unit change in seaweed production. Data were checked to ensure that the assumptions of linear regression in terms of distribution and homogeneity [54]. Regressions included only years where commercial seaweed farming began consistently so that initial low years prior to wide scale commercial production did not confound the results. Outliers, defined as data points diverging more than three standard deviations from the mean (there was 1 in the analysis), were also removed from the analysis. Slopes of the regression line were interpreted as a rate of increase relative to seaweed production in order to compare the relationship between siganid catch and seaweed production against other reef fish (excluding siganids) and seaweed production, which acted as a *de facto* control for increased fishing pressure across all reef fish. Differences between slopes were tested using a *t* test [22,54].

## Results

### Local analysis

**Fig 2:**
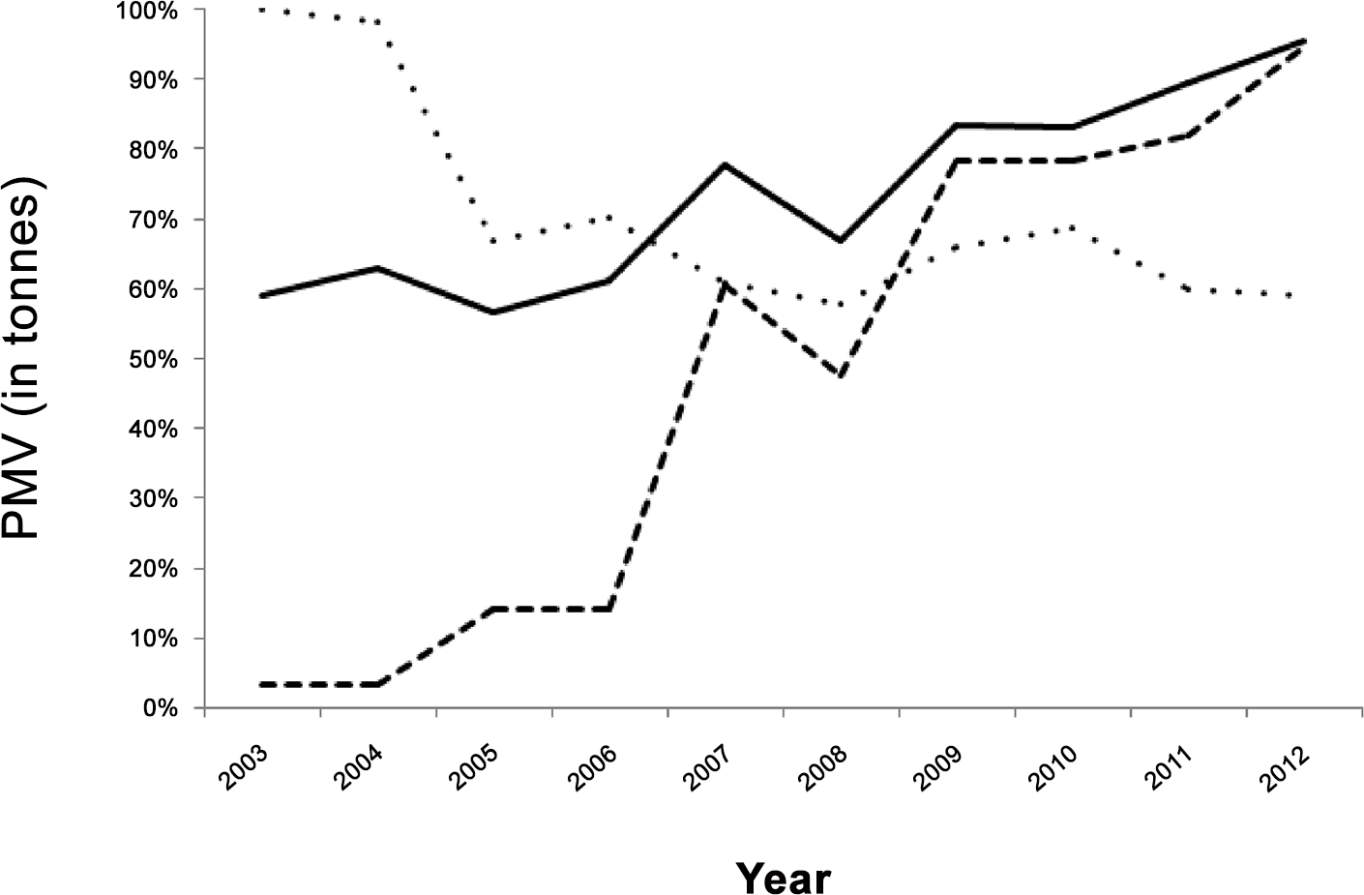
Temporal trends in seaweed production (solid line), siganid catch (dashed line) and reef fish catch (dotted line) as a percentage of maximum value (PMV) for the Bohol Province, Philippines.

Reports from the province of Bohol to the PBAS revealed that widescale farmed seaweed production for both elkhorn (*K*. *alvareezi*) and eucheuma (*E*. *spinosum*) began in 1995. Over a twelve-year time period, production increased steadily from 74,755 tonnes per annum in 2002 to 126,551 in 2011, with the exception of 2008 where mean annual production declined to 84,924 tonnes (Fig 2). A local survey of reef fish including siganids conducted by the Bohol office of the PBAS recorded landings for the same time period peaked at 825.8 tonnes in 2011, which coincided with the maximum production in seaweeds (Fig 2).

**Fig 3:**
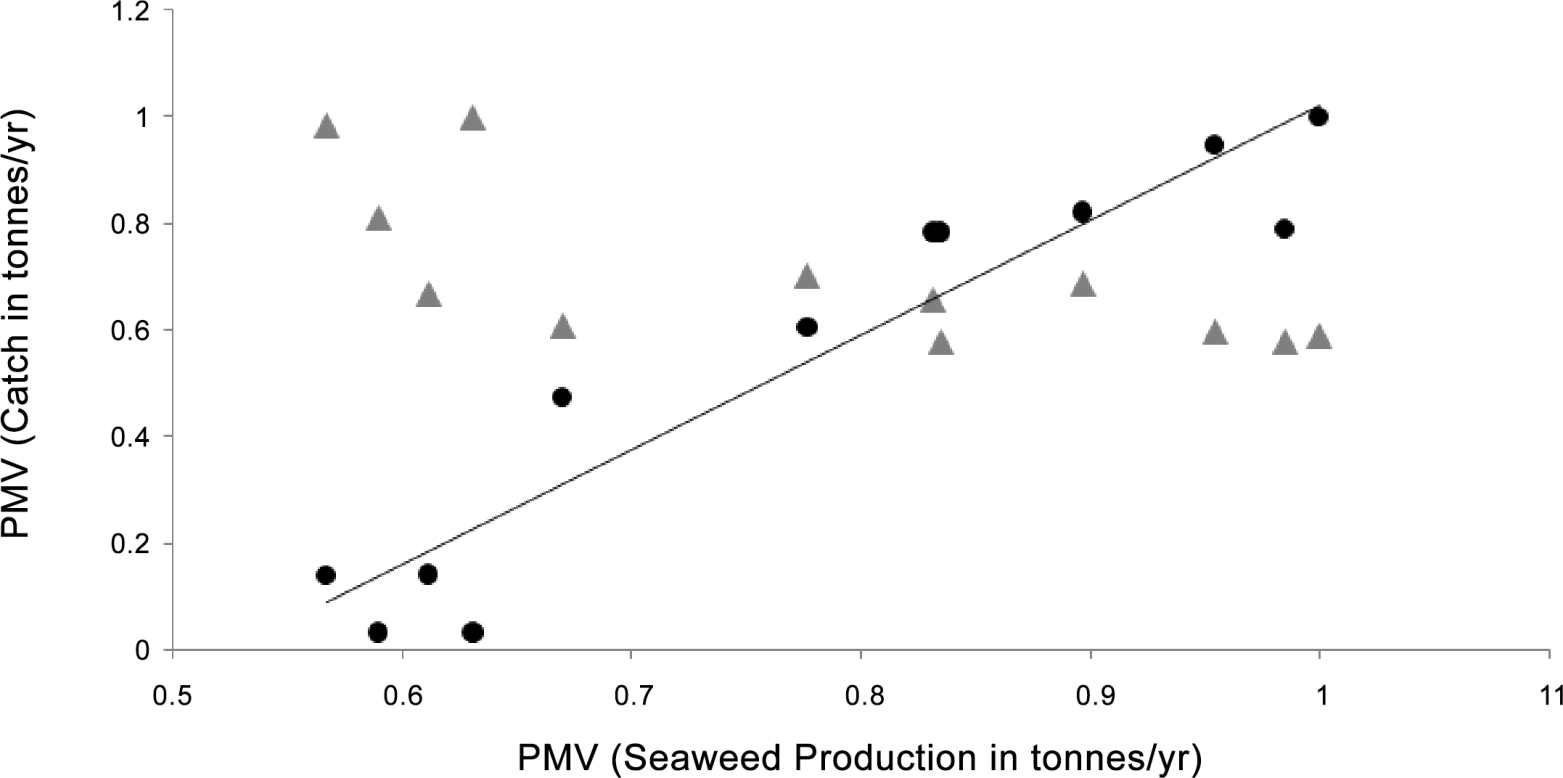
Comparison of the relationships between siganid catch (circles) and reef fish catch (triangles) vs. seaweed production, with all values calculated as a percentage of the maximum value (PMV) in tonnes for Bohol Province, Philippines over the period 2002–2012

Reef fish landings for the same time frame were highest in 2004 at 80170 tonnes (Fig 2). The relationship between siganid catch and seaweed production was significant and positive (p = 2.77 E-06, R^2^ = 0.89, n = 12), however there was no significant relationship between reef fish catch and seaweed production (p = 0.10, R^2^ = 0.50, n = 12): reef fish catches initially decreased relative to seaweed production and in general were steady or declining with rising seaweed production (Table 1; Fig 3). We did not compare the slopes given the non-significant relationship for reef fish catch and seaweed production (Table 1; Fig 3).

**Table 1:** Regression statistics for siganid and reef fish catches as a function of seaweed production respectively, including the estimated slope, intercept, coefficient of determination (R2) and p values. Where both relationships are positive and significant (p<0.05), slopes were compared with a t test with corresponding t-values (t), degrees of freedom (df) and p values presented. NT indicates no test. Results are presented for the regional analysis (Bohol) and for the three regions: Southeast Asia (Indonesia, Malaysia, and the Philippines), Africa (Tanzania and Zanzibar), and the Western Pacific (Fiji).

Country/Province	Siganids				Reef fish				Comparison of Slopes		
	slope	intercept	R2	p	slope	intercept	R2	p	t	df	p
Bohol	2.06	-1.05	0.89	**2.77 E-06**	-0.66	1.22	0.50	0.10			NT
Indonesia	0.80	0.26	0.78	**0.004**	0.26	0.59	0.53	**0.006**	3.97	12	<0.001
Malaysia	0.33	0.68	0.55	**0.01**	0.24	0.45	0.72	**0.002**	6.36	16	<0.001
Philippines	0.44	0.43	0.65	**5.5 E-06**	0.20	0.42	0.82	**1.0 E-06**	3.20	40	<0.002
Tanzania	-0.41	0.82	0.40	**0.001**	0.13	0.38	0.18	**0.04**			NT
Zanzibar	-0.01	0.70	0.00	0.58	-0.29	0.74	0.41	**0.03**			NT
Fiji	0.37	0.25	0.37	0.09	0.12	0.19	0.11	**0.008**			NT

### Regional analyses

Fishbase lists 23 species of Siganid for Southeast Asia, Africa, and the Western Pacific (Table 2). Nine species are common to Southeast Asia and the Western Pacific, while only two: *Siganus argenteus* and *Siganus stellatus* are confirmed between Southeast Asia and Africa, with another three species, *Siganus guttatus*, *Siganus rivulatus* and *Siganus sutor* listed as possibly shared but unconfirmed.

**Table 2:** The 23 Siganid species for six countries used in the analysis and their diets as listed on Fishbase, where P is a possible but unconfirmed sighting.

Siganid species	Zanzibar	Tanzania	Fiji	Philippines	Indonesia	Malaysia	Diet
*Siganus argenteus*	✓	✓	✓	✓	✓	✓	Plants/Zoobenthos
*Siganus canaliculatus*			✓	✓	✓	✓	Plants/Zoobenthos
*Siganus corallinus*			✓	✓	✓	✓	Plants/Zoobenthos
*Siganus doliatus*			✓	✓	✓	✓	Plants
*Siganus fuscescens*				✓	✓	✓	Plants/Detritus/Zoobenthos
*Siganus guttatus*	**P**	**P**		✓	✓	✓	Plants/Zooplankton
*Siganus javus*				✓	✓	✓	Plants/Detritus/Zoobenthos
*Siganus labyrinthodes*					✓		Plants
*Siganus lineatus*				✓	✓	✓	Plants
*Siganus luridus*	✓	✓					Plants
*Siganus magnificus*					✓		Plants/Zooplankton
*Siganus puellus*				✓	✓	✓	Zoobenthos
*Siganus punctatissimus*			✓	✓	✓	✓	Plants
*Siganus punctatus*			✓	✓	✓	✓	Plants
*Siganus rivulatus*	✓	✓		**P**			Plants
*Siganus spinus*			✓	✓	✓		Plants
*Siganus stellatus*	✓	✓			✓	✓	Plants/Zooplankton
*Siganus sutor*	✓	✓			**P**	**P**	Plants
*Siganus unimaculatus*				✓			Plants/Zooplankton
*Siganus uspi*			✓				Plants/Zooplankton
*Siganus vermiculatus*			✓	✓	✓	✓	Plants
*Siganus virgatus*				✓	✓	✓	Plants/Zooplankton
*Siganus vulpinus*				✓	✓	✓	Plants/Zooplankton

**Fig 4:**
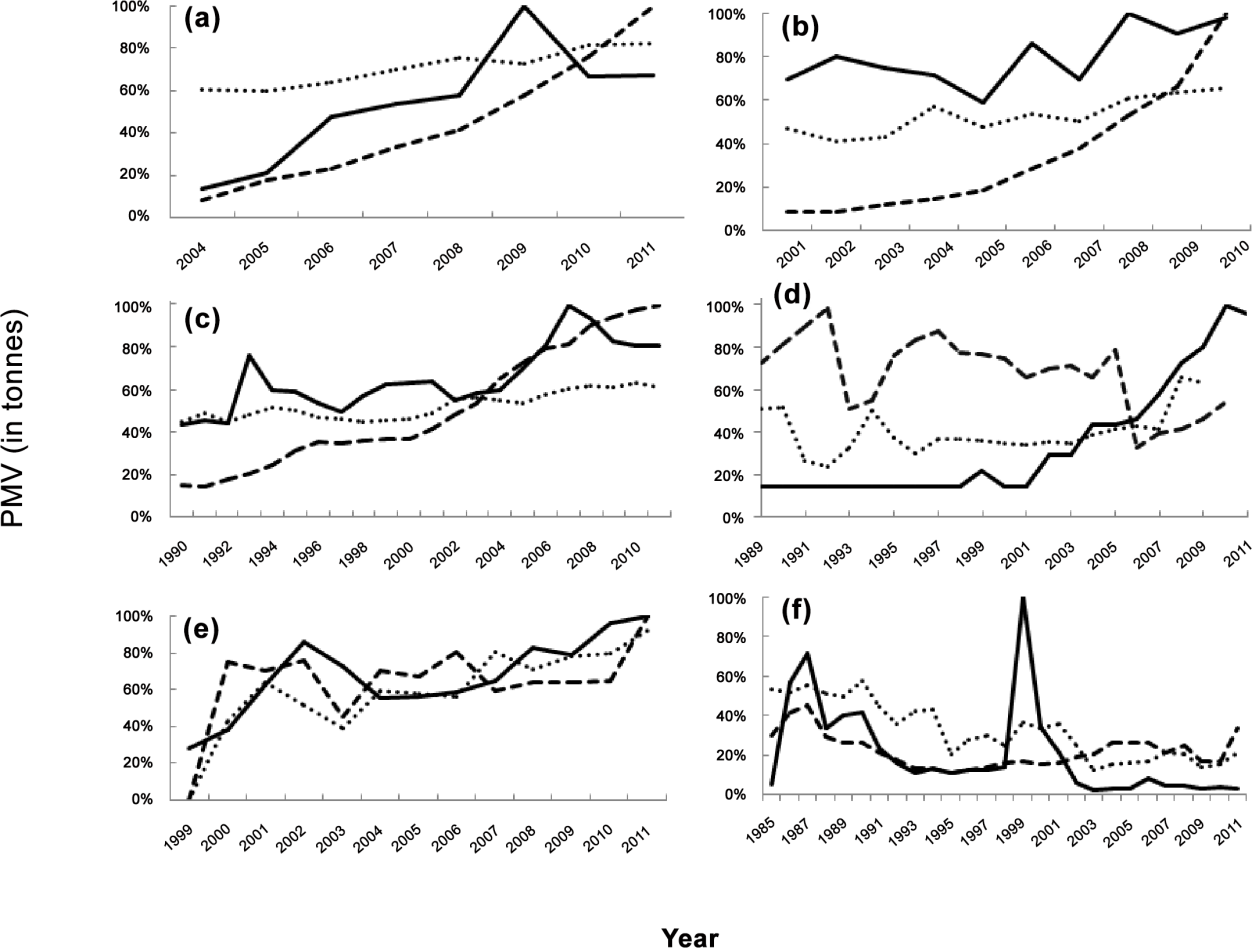
Temporal trends in seaweed production (solid line), siganid catch (dashed line) and reef fish catch (dotted line) as a percentage of maximum value (PMV) for the focal countries in each of the three regions: Southeast Asia (a) Indonesia, (b) Malaysia, (c) the Philippines; Africa (d) Tanzania and (e) Zanzibar; and the Western Pacific (f) Fiji.

**Fig 5:**
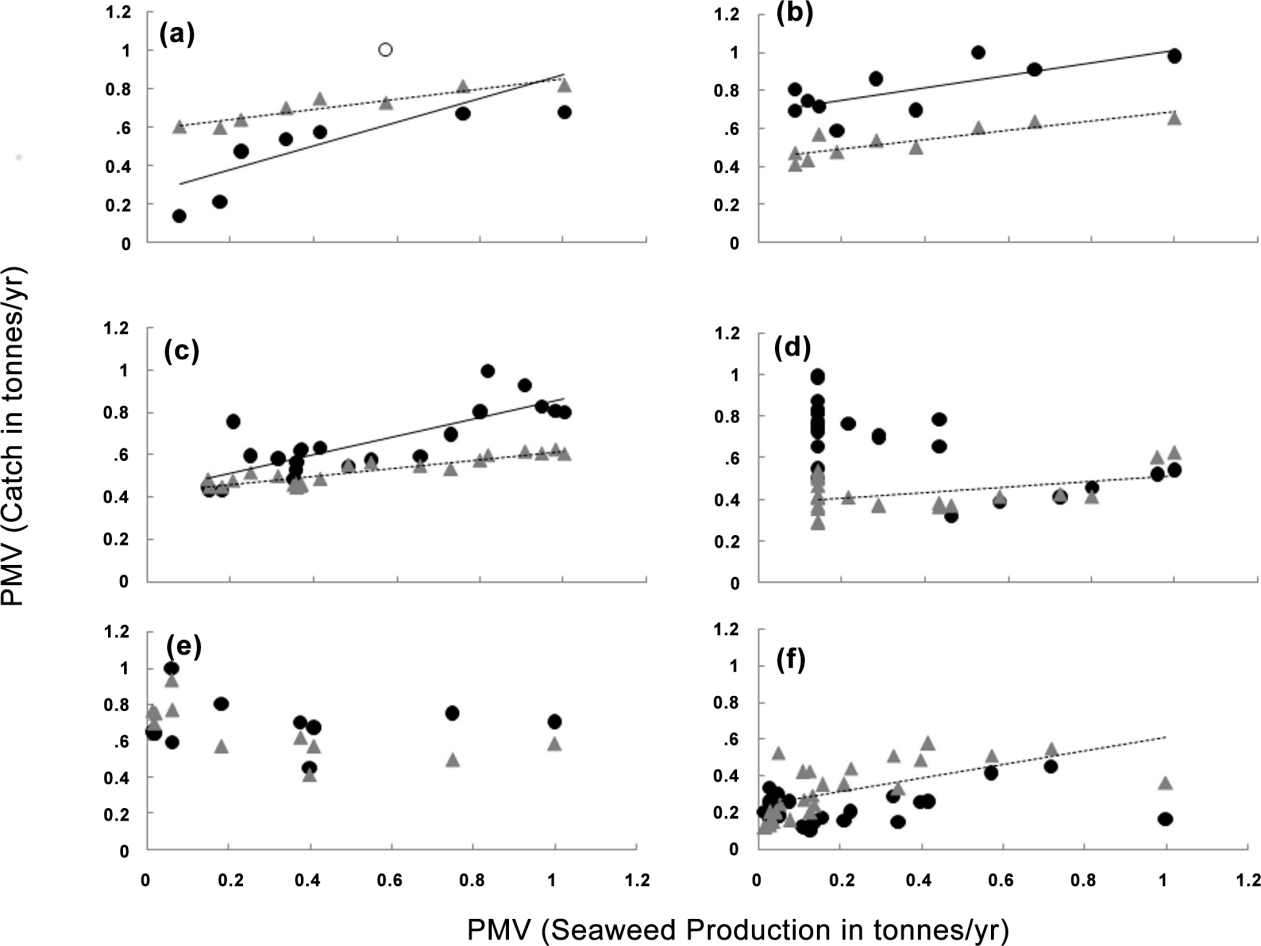
Comparison of the relationships between siganid catch and seaweed (circles) and reef fish catch (triangles) vs. seaweed production, with all values calculated as a percentage of maximum value (PMV) for three regions: (Southeast Asia, Africa, and the Western Pacific) (a) Indonesia, (b) Malaysia, (c) the Philippines (d) Tanzania (e) Zanzibar; and (f) Fiji. *Values with an open circle are outliers were removed from analysis but presented on the figure.

Siganid landings in Southeast Asia (Indonesia, Malaysia and the Philippines) increased at a faster rate relative to seaweed production than did reef fish landings as a proportion of the maximum value (PMV) (Fig 4). Further, Siganid catch was significantly correlated with seaweed production (Table 1; Fig 5). The other regions showed no consistent patterns.

### Southeast Asia

In Indonesia, reports to the FAO were initiated in 1950 under the category of “red algae” (Fig 4). In 2000, this category was made redundant and production was instead allocated to “Eucheuma nei” and “Gracilaria spp”. Eucheuma nei was dominant and accounted for 92% of total red algae production on average (range: 86%-97%), with Gracilaria spp. tending to become more important through time. Given the long time series available for the combined production of Eucheuma nei and Gracilaria spp, we summed the two between 2000 and 2011 for a combined value comparable to that reported from 1950 to 1999. Seaweed production varied from approximately 10 tonnes in 1950 to 5,170,201 tonnes in 2011, exhibiting a sharp increase onward of the 1990s (Fig 4). Reporting of reef fish generally commenced in 1950, but siganid landings were only reported from 2004 to 2011, showing an annual rate of catch increase of 8% per annum, with some suggestion of a decline in the last two years (Fig 4). Mean landings of reef fish in terms of PMV increased steadily at 2% per annum from 1975 (Fig 4). There were significant positive relationships between siganid catch and farmed seaweed (p = 0.004; R^2^ = 0.78, n = 8) (Table 1; Fig 5), as well as between reef fish catch and farmed seaweed (p = 0.0006; R^2^ = 0.88, n = 8), (Table 1; Fig 5). Comparison of the slopes showed siganid catch increased more quickly relative to seaweed production than did reef fish catch (t = 3.97; p<0.001) (Table 1; Fig 5).

In Malaysia, reports to the FAO began in 2001 for both Spiny Eucheuma and Elkhorn with the latter accounting for about 96% of the production over this period. Annual production varied from approximately 863 and 18,000 tonnes in 2001 to a peak of 7,892 (2010) and 239,450 (2011) for Spiny Eucheuma and Elkhorn respectively (Fig 4). Both taxa showed rapid increases in production averaging approximately 9–10% per annum over this period. Reporting of reef fish generally commenced in 1950, and siganid landings were reported from 1982 to 2011 during which period there was an approximate 1% increase per annum in siganid landings. Mean catch of other reef fish increased steadily at 1.7% per annum from 1975 (Fig 4). There was a significant positive relationship between siganid catch and seaweed production (p = 0.01; R^2^ = 0.55; n = 10) (Table 1; Fig 5) and between reef fish catch and seaweed production, (p = 0.002; R^2^ = 0.72; n = 10) (Table 1; Fig 5). Comparison of the slopes showed siganid catch increased more quickly relative to seaweed production than did reef fish catch (t = 6.61; p<0.001)(Table 1; Fig 5).

In the Philippines, reports to the FAO began in 1965 for Elkhorn, 1974 for Spiny Eucheuma, and in 2002 for Gracilaria. Gracilaria is reported at very low levels, accounting for typically less than 0.1% of the combined production of the three seaweeds. Production of Elkhorn varied from 1,000 tonnes to 1,697,682 tonnes while Spiny Eucheuma ranged from 3,000 tonnes to 136,183 tonnes per annum, and Gracilaria from 389 to 2479 (Fig 4). Elkhorn showed a steady rise from 1980 with two distinct peaks in 1980 and 1996. Spiny Eucheuma showed a sharp increase in production from 2000 at a rate of approximately 5% per annum. Reporting of reef fish generally commenced in 1950, with siganid catches reported from 1963 to 2011, during which period there was an approximate 2% increase per annum (Fig 4). Mean landings of other reef fish increased steadily at less than 1% per annum over the same period (Fig 4). There were strong significant positive relationships between siganid catch and farmed seaweed production, and between reef fish catch and seaweed production (p = 5.50 E-6; R^2^ = 0.65; n = 22, and p = 1.0 E-06; R^2^ = 0.81; n = 22, respectively) (Table 1; Fig 5). Comparison of the slopes showed siganid catch increased more quickly relative to seaweed production than did reef fish catch (t = 3.20; p<0.002) (Table 1; Fig 5).

### Africa

In Tanzania, reports to the FAO began in 1989 under the heading of “Eucheuma species nei” (Fig 4). Production varied from approximately 1,000 tonnes per annum in 1989 to 6,885 tonnes per annum in 2010, exhibiting a sharp increase in production of approximately 8% per annum from 2001 (Fig 4). Reporting of reef fish generally commenced in 1973 with a large increase in siganid landings between 1989 and 2005, after which landings decreased substantially (Fig 4). There was a significant but negative correlation between siganid catch and farmed seaweed production (p = 0.0001, R^2^ = 0.40; n = 23) while reef fish catch was significantly and positively correlated to seaweed production (p = 0.04; R^2^ = 0.18; n = 23) (Table 1; Fig 5).

In Zanzibar, reports to the FAO began in 1990 under the heading of “Spiny Eucheuma” (Fig 4). Production varied from 8,080 tonnes per annum in 1990 to 129,779 tonnes per annum in 2011, exhibiting a general steady increase of approximately 4% per annum (Fig 4). Reporting of reef fish commenced in 2000 with no clear trends in siganid landings: catches ranged from 710 tonnes per annum in 2003 to 1573 tonnes per annum in 2011 with a mean of 1096 tonnes per annum (±207 SD) and no trends through time. Mean landings of other reef fish generally increased between 2000 and 2011 at a rate of 4% per annum (Fig 4). There was no significant relationship between siganid catch and farmed seaweed production (p = 0.58; R^2^ = 0.005; n = 12), and reef fish catch and seaweed production though significant, were negatively correlated (p = 0.03; R^2^ = 0.41; n = 12) (Table 1; Fig 5).

### Western Pacific

In Fiji, reports to the FAO on farmed seaweed production began in 1985 under the heading of “Eucheuma nei” (Fig 4). Production varied from 250 tonnes per annum in 2003 to 15,090 tonnes per annum in 1999. There was a strong boom-and-bust cycle with two major peaks in 1987 and 1999. Annual production was highly variable until 1989, when it declined to, and stabilised at a mean value of approximately 590 tonnes per year (±260 SD). Reported landings of siganids ranged between 62 and 595 tonnes per annum, reaching a general plateau from 1991 to 2011 of 112 tonnes per annum (±34.9 SD). Siganid landings peaked in 1980, six years prior to the first peak in farmed eucheuma. Mean landings of other reef fish generally declined from the 1980s onwards. There were no significant relationships between siganid catch and farmed eucheuma production (p = 0.09, R^2^ = 0.11, n = 27) although there was a significant positive relationship between reef fish catch and seaweed production (p = 0.008, R^2^ = 0.37, n = 27) over the period that eucheuma production was reported (1985–2011) (Table 1; Fig 5).

## Discussion

At the regional level in Bohol, our study documented a positive relationship between siganid catch and the production of farmed seaweed with siganid catches increasing more rapidly. This lends empirical support to the idea that more abundant food supplies may increase production of some herbivorous reef fish [2,22–25,55,56]. Such a derived benefit appears to have occurred despite the elevated levels of habitat fragmentation driven by the rapid expansion of the seaweed farming industry [1,2,22,25,37–39] and within the context of a complex mosaic of anthropogenic use in the generally degraded seascapes of Danajon Bank region [20,44]. Reefs in the area were already highly degraded from a host of anthropogenic activities before the addition of seaweed farming. However, clearing associated with the establishment of farms would serve to remove the living coral and rubble alike, along with constituent seaweeds, in order to reduce entanglement of the monolines used in farms [44,57]. However, in spite of the additional homogenisation of the substrate associated with farms, they would also serve to introduce a food source for the herbivorous rabbitfish and it is therefore possible that in this context, the establishment of seaweed farms fosters increased rabbitfish catch.

Fishing effort is a spatially and temporally heterogeneous process that generally increases as a function of human population size [50–52]. Therefore it was necessary to establish a control for inherent differences in fishing effort. The interpretation of the positive correlation between siganid catch and seaweed production as evidence of a seaweed-derived benefit to siganids, is based on the use of the reef fish /seaweed production relationship as a proxy for fishing effort. In Bohol, the relationship between reef fish and seaweed production is flat, relative to the doubling of siganid catch per unit increase in seaweed production. In context, within the same region, fisheries catch per unit effort is declining [47] due to a declining resource base. The reef fish comparison may be inappropriate if the set of species used in the comparison are unrepresentative of general fishing effort, but care was taken to incorporate species subject to similar fishing techniques and found on similar habitats. It may also be inappropriate if there has been a shift in effort towards siganids over this period. This is unlikely as there is a long history of siganid extraction in the region [47], particularly in light of the region’s depleted state since the 1970s. The comparison does suggest that siganid catches are increasing disproportionately quickly relative to reef fish catches that are in decline, which provides confidence in the use of reef fish catch as a control.

We observed similar patterns regionally in Southeast Asia, where strong correlations were found between siganid catch and seaweed production, and where these relationships showed more rapid rates of increase than those based on reef fish and seaweed production. The strongest correlations between siganids and farmed red seaweeds were observed in Southeast Asia. Given the Philippines and Indonesia are respectively ranked 2^nd^ and 3^rd^ for global production of carrageenophytes, and Malaysia 7^th^, [1], this suggests a pattern of global significance. Such patterns were not, however, apparent in either Africa or the Western Pacific. Differences between seaweed production in Africa and the Western Pacific, as opposed to Southeast Asia, lie both in the duration and continuity of farming, which could have significant implications for the establishment of farmed seaweed as a food source for reef fish. In Fiji, for instance, the recurrent destruction of farming operations by typhoons has discouraged farmers from investing in infrastructure [45,58], and as a result, seaweed farming has been re-introduced on at least three occasions since the 1970s, typically on a small scale. Furthermore, fluctuating world market prices, high transportation costs to remote farming sites and an absence of local processing infrastructure all make seaweed farming less attractive than traditional fishing to many Fijians [45,58].

Similarly, in Africa, commercial seaweed farming has been both slow to establish and inconsistent in its application due to several factors, including (a) the failure of an economically valuable species of carrageenophyte, *K*. *alvarezii* (Elkhorn moss), and (b) societal and cultural changes associated with increased farming activities [59]. For example, *K*. *alvarezii*, the most profitable seaweed species, is now failing to grow in areas where it was previously cultivated due to changes in environmental conditions. These changes include warmer seas, epiphytism, and fouling [60,61]. Additionally, while initially promoted as a tool for coastal development particularly for women in Africa, further studies have shown that many women were abandoning seaweed farm because of health concerns [59,62,63] as well as in response to negative perceptions of the benefits of farming compared to the additional workload [62], and pressure over the associated cultural and societal changes brought by increased farming activities [59,64].

The scale of commercial seaweed farming may be another contributing factor to differences between Southeast Asia and Africa and the Western Pacific. Africa and the Western Pacific make relatively small contributions to worldwide production (< 1%) [1]. In Fiji, seaweed farming has occurred on a fairly small scale, with the maximum export occurring close to the inception of commercial production in 1987 and with a peak export of only 10,850 tonnes [1]. Further, in the 26 years since the commencement of commercial seaweed farming in Fiji, there has only been one increase in total production in six years (in 2000), and the overall trend has been one of general decline [1]. In both Tanzania and Zanzibar, maximum seaweed production was 129,000 and just over 100,000 tonnes respectively. These levels are orders of magnitude lower seaweed production levels in Southeast Asia. It is important to recognise that the scale of seaweed farming is to some degree a function of available habitat for farms. There is much less reef in Tanzania, Zanzibar and Fiji than in the Southeast Asian countries, 28% vs 4.8% of world’s reefs [1]. Where seaweed farming occurs in Southeast Asia, it tends to be concentrated: seaweed production for Indonesia, Malaysia and the Philippines exceeds 101 t•km^-^2, 72 t•km^-^2, and 70 t•km^-^2, based on total reef area by nation[65], and regions such as Bohol support intense production [47]. In Africa and the Western Pacific seaweed production for Tanzania and Fiji was only 2 t•km^-^2 [65], with farming occurring in low volumes compared to the scale possible based on available reefs. It may be that under these conditions, seaweed production does not increase to a threshold sufficient to support increased siganid catches. The exception is Zanzibar, which has relatively high seaweed production relative to reef area [65]. However, the comparison is inappropriate for Zanzibar as most seaweed production here occurs on sand flats and not shallow coral reefs. The implication is that in locations with low areal coverage, reef fish would be much less likely to encounter and subsequently benefit from farmed seaweed.

Differences in the relationships between siganids, reef fish, and seaweed production in Southeast Asia and Africa may also reflect differences in the ecology and feeding strategies of the siganids found in these regions [34,35,66–69]. For example, in Bohol, *S*. *canaliculatus* is the major siganid targeted by fisheries. It tends to settle directly on algal beds rather than coral reefs and consumes macroalgae [34], and therefore may be particularly well adapted to taking advantage of the implementation of seaweed farming. In contrast, *Siganus sutor*, common in the Indian Ocean Region and east Africa, while known to settle in algal beds like *S*. *canaliculatus*, exhibits a dietary preference for turf algae [70–73], suggesting that seaweed farms would not provide the same dietary benefits for *S*. *sutor* as they would for *S*. *canaliculatus*. In the absence of taxonomic resolution within the FAO global database, regional studies will help elucidate the relative importance of seaweed farming in terms of the provision of shelter vs. the provision of additional food sources.

The differences in feeding ecology of the main targeted species between the two regions may also underpin the lack of relationship between siganid catches and seaweed production in the Eastern Indian Ocean. Siganid fisheries for *S*. *sutor* in the Indian Ocean appear to be enhanced by the presence of algal dominated degraded reefs [70–73], reflecting *S*. sutor’s dietary preference for turf algae within these systems. As seaweed farms result in the clearing of turf algae, seaweed farming may present a dietary penalty for *S*. *sutor* rather than a benefit as it does for *S*. *canaliculatus*. Such a scenario would imply that the effects of seaweed farming may vary depending on location and species, and highlights the need for further investigation into both the ecological and dietary strategies of affected fish assemblages.

Another possibility is that siganid catch may not be a direct result of seaweed farming itself, but an effect of algal domination as a result of coral reef degradation present where seaweed farming tends to occur, but initiated before commercial seaweed farming. Coral reefs in Southeast Asia show significantly higher levels of human impact from a variety of activities than either Fiji or Africa [19], and degraded reefs tend to be dominated by macroalgae [74]. As was the case in the central Philippines where the consumption of farmed red seaweeds by siganids initially appeared as a direct food subsidy (Hehre & Meeuwig in review), farms in Southeast Asia tend to be situated across wide expanses of algal dominated reefs that have subsequently been cleared for farms, leaving farmed seaweed as a replacement for areas that would have otherwise contain wild food items. Following such a system shift from algal dominated coral reefs to seaweed farms, farmed seaweed may affect fish populations in two ways. Farms may provide a replacement food source where farms have been established by clearing reefs. In this case seaweed farms, while increasing siganid catches do so only because other seaweeds have been cleared as a result of their implementation thereby resulting in either increases or maintenance of herbivorous fish. Second, farms may serve to physically concentrate populations of dependent fish, which in turn may facilitate their capture. FAO data alone do not encapsulate this information nor does it allow us to disentangle these two processes without additional surveys. It is therefore difficult to confirm whether seaweed farming provides a true subsidy to fish or whether the benefits derived from the implementation of commercial seaweed production come as a result of a decrease in other available food items.

In the face of declining returns from fisheries depleted by over-extraction, destructive fishing practices, and habitat degradation over an extended period of time [45], the potential for an expanding seaweed farming industry to enhance the productivity of a valuable food fish, the siganid, is potentially important to both artisanal fishers and seaweed farmers alike. Over the last two decades, seaweed farming has grown worldwide and become an important commodity on the world market that generates significant socio-economic benefits for marginalized coastal communities in developing countries. Higher continuity, less sporadic production and higher volumes of seaweed production may explain why siganid catch increased disproportionately faster than reef fish catches in Southeast Asia when compared to Africa and the South Pacific, and therefore, this correlative study demonstrates the potential for seaweed farming to increase siganid catch.

## Supporting Information

S1 Table Common names of demersal fish from six countries; Fiji, Indonesia, Malaysia, Philippines, Tanzania, and Zanzibar, as retrieved from the FAO database and their corresponding family names for Bohol Province and six countries. (DOCX)
