# Supplementary material for: A Global Analysis of the Relationship between Farmed Seaweed Production and Herbivorous Fish Catch
Source: PLoS One. 2016 Feb 19;11(2):e0148250. doi: 10.1371/journal.pone.0148250 (PMC4760753; doi:10.1371/journal.pone.0148250)
Supplement: S1 Table — (DOCX) [file pone.0148250.s001.docx]

| FAO (common name) | Family | Bohol | Fiji | Indonesia | Malaysia | Philippines | Tanzania | Zanzibar |
| --- | --- | --- | --- | --- | --- | --- | --- | --- |
| Anchovies | Engraulidae | x |  |  |  |  |  |  |
| Batfishes | Ephippidae |  |  |  |  | x |  |  |
| Bigeyes | Priacanthidae |  |  | x | x |  |  |  |
| Bombay-duck | Synodontidae |  |  | x | x |  |  |  |
| Cardinalfishes | Apogonidae |  | x |  |  |  |  |  |
| Chocolate hind | Serranidae |  |  | x |  |  |  |  |
| Conger eels | Congridae |  |  |  |  | x |  |  |
| Croakers, drums | Sciaenidae |  |  | x | x |  |  |  |
| Daggertooth | Muraenesocidae |  |  |  | x |  |  |  |
| Eeltail catfishes | Plotosidae |  |  |  | x |  |  |  |
| Emperors | Lethrinidae |  | x | x | x |  | x | x |
| Flatfishes | Paralichthyidae |  |  | x | x | x |  |  |
| Flatheads | Platycephalidae |  |  |  | x |  |  |  |
| Fusiliers | Caesionidae | x |  | x | x | x |  |  |
| Glassfishes | Gerridae |  |  |  |  | x |  |  |
| Goatfishes | Mullidae | x | x | x | x | x |  | x |
| Gobies | Gobiidae |  |  |  |  | x |  |  |
| Groupers | Serranidae | x | x |  | x | x | x | x |
| Grunts, sweetlips | Lethrinidae |  |  | x | x |  |  |  |
| Hairtails, scabbardfishes | Trichiuridae | x |  | x | x | x |  |  |
| Indian halibut | Psettodidae |  |  | x |  |  | x |  |
| Indo-Pacific mackerel | Scombridae | x |  |  |  |  |  |  |
| Indo-Pacific tarpon | Megalopidae |  |  |  | x | x |  |  |
| Largeeye breams | Lethrinidae |  | x |  |  |  |  |  |
| Lizardfishes | Synodontidae |  |  | x | x | x |  |  |
| Mangrove red snapper | Lutjanidae |  |  |  | x |  |  |  |
| Mojarras, silver biddies | Gerridae |  | x |  | x | x |  |  |
| Monocle breams | Nemipteridae |  |  |  | x |  |  |  |
| Moonfish | Lampridae |  |  |  |  | x |  |  |
| Mullets | Muglidae | x | x | x | x | x | x | x |
| Parrotfishes | Scaridae | x |  |  | x |  |  | x |
| Ponyfishes, Slipmouths | Leiognathidae | x | x | x |  | x |  |  |
| Porgies, seabreams | Lethrinidae | x |  |  |  | x |  |  |
| Big-eyed scad | Carangidae | x |  |  |  |  |  |  |
| Sardines | Clupeidae | x |  |  |  |  |  |  |
| Scats | Scatophagidae |  |  |  |  | x |  |  |
| Sea catfishes | Plotosidae |  |  | x | x | x | x |  |
| Sillago-whitings | Sillaginidae |  |  |  | x | x |  |  |
| Snappers | Lutjanidae | x | x | x | x | x |  | x |
| Spinefeet, rabbitfish | Siganidae | x | x |  | x | x |  | x |
| Spotted sicklefish | Drepaneidae |  |  |  | x | x |  |  |
| Surgeonfishes | Acanthuridae |  | x |  |  | x |  |  |
| Threadfin breams | Nemipteridae | x |  | x | x | x | x |  |
| Threadfins, tasselfishes | Polynemidae |  |  |  | x | x |  |  |
| Tonguefishes | Cynoglossidae |  |  |  | x |  |  |  |
| Triggerfishes, durgons | Balistidae |  | x |  | x |  |  |  |
| Wrasses, hogfishes | Labridae |  | x |  | x | x | x |  |
|  |  |  |  |  |  |  |  |  |

S1 Table. Common names of demersal fish from six countries; Fiji, Indonesia, Malaysia, Philippines, Tanzania, and Zanzibar, as retrieved from the FAO database and their corresponding family names for Bohol Province and six countries
